# Toward Studying Music Cognition with Information Retrieval Techniques: Lessons Learned from the OpenMIIR Initiative

**DOI:** 10.3389/fpsyg.2017.01255

**Published:** 2017-08-03

**Authors:** Sebastian Stober

**Affiliations:** Machine Learning in Cognitive Science Lab, Research Focus Cognitive Sciences, University of Potsdam Potsdam, Germany

**Keywords:** music cognition, music perception, music information retrieval, deep learning, representation learning

## Abstract

As an emerging sub-field of music information retrieval (MIR), music imagery information retrieval (MIIR) aims to retrieve information from brain activity recorded during music cognition–such as listening to or imagining music pieces. This is a highly inter-disciplinary endeavor that requires expertise in MIR as well as cognitive neuroscience and psychology. The OpenMIIR initiative strives to foster collaborations between these fields to advance the state of the art in MIIR. As a first step, electroencephalography (EEG) recordings of music perception and imagination have been made publicly available, enabling MIR researchers to easily test and adapt their existing approaches for music analysis like fingerprinting, beat tracking or tempo estimation on this new kind of data. This paper reports on first results of MIIR experiments using these OpenMIIR datasets and points out how these findings could drive new research in cognitive neuroscience.

## 1. Introduction

Music Information Retrieval (MIR) is a relatively young field of research that has emerged over the course of the last two decades. It brings together researchers from a large variety of disciplines who—in the broadest sense—investigate methods to retrieve and interact with music information. As the MIR community has grown, research questions also have become more diverse. The different kinds of data considered in MIR now comprise, for instance, symbolic representations, audio recordings, sheet music, playlists, (social) web data such as reviews or tweets, and usage meta-data.

As a very recent development, MIR researchers also have started to explore ways to detect and extract music information from brain activity recorded during listening to or imagining music pieces–a sub-field of MIR introduced as Music Imagery Information Retrieval (MIIR) in Stober and Thompson (2012). In the long term, research in this direction might lead to new ways of searching for music along the line of existing MIR approaches that, for instance, allow query by singing, humming, tapping, or beat-boxing. Inspired by recent successes in reconstructing visual stimuli (Miyawaki et al., 2008; Nishimoto et al., 2011; Cowen et al., 2014) and even dream imagery (Horikawa et al., 2013), it might eventually be possible to even reconstruct music stimuli from recorded brain activity.

In a broader context, Kaneshiro and Dmochowski (2015), for instance, mention transcription, tagging and annotation, audience following, and portable MIR applications as possible scenarios that could benefit from neuroimaging data such as EEG. Findings from MIIR can further support the development of Brain-Computer Interfaces (BCIs) that facilitate interaction with music in new ways beyond basic search–such as Brain-Computer Music Interfaces (BCMIs) used to generate and control music (Miranda and Castet, 2014). Finally and most importantly, this paper aims to motivate an MIIR-driven approach to music cognition research that can lead to new insights about on how the human brain processes and encoders music.

The challenge of retrieving music information from recordings of brain activity can in principle be approached in the following naïve way: One could argue that as the brain processes perceived music or recreates this experience in imagination, it generates a transformed representation which is captured—to some extend—by the recording equipment. Hence, the recorded signal could in principle be seen as a mid-level representation of the original music piece that has been heavily distorted by two consecutive black-box filters—the brain and the recording equipment. This transformation involves and intermingles with several other brain processes unrelated to music perception and is further limited by the capabilities of the recording equipment which might additionally introduces signal artifacts.

This setting calls for sophisticated signal processing techniques, ideally developed in an intense interdisciplinary collaboration between MIR researchers and neuroscientists. In order to facilitate such a collaboration and contribute to new developments in this emerging field of research, the first OpenMIIR dataset was released as public domain in 2015 (Stober et al., 2015b). This article summarizes work over the course of a year since its publication. To this end, a brief overview of the dataset is provided in section 2. as well as related research in section 3. As the main part of this paper, section 4 covers our experiments. This is followed by a discussion in section 5. Finally, we draw conclusions and point out directions for future work in section 6.

## 2. The OpenMIIR dataset

The OpenMIIR dataset (Stober et al., 2015b) comprises Electroencephalography (EEG) recordings taken during music perception and imagination.^1^ These data were collected from 10 subjects who listened to and imagined 12 short music fragments—each 7–16 s long—taken from well-known pieces. EEG was chosen as recording technique because it is much more accessible to MIR researchers than Magnoencephalography (MEG) and functional Magnetic Resonance Imaging (fMRI), with more and more affordable consumer-level devices becoming available. Furthermore, EEG has a good temporal resolution that can capture how music perception and imagination unfold over time and allows for analyzing temporal characteristics of the signal such as rhythmic information. The stimuli were selected from different genres and systematically span several musical dimensions such as meter, tempo, and the presence of lyrics. This way, various retrieval and classification scenarios can be addressed. As shown in Table 1, there are 3 groups with 4 stimuli each.

1. Stimuli 1–4 are from recordings of songs where a singing voice (lyrics) is present.
2. Stimuli 11–14 are from different recordings of the same songs as stimuli 1–4. These recordings do not contain a singing voice. Instead, the melody is played by one or more instruments.
3. Stimuli 21–24 are from recordings of purely instrumental pieces that do not have any lyrics and thus it is not possible to sing along.

**Table 1:** Information about the tempo, meter, and length of the stimuli (without cue clicks).

**ID**	**Group**	**Name**	**Meter**	**Length**	**Tempo in beats per minute (BPM)**
1	Songs recorded with lyrics	Chim Chim Cheree	3/4	13.3 s	212
2		Take Me Out to the Ballgame	3/4	7.7 s	189
3		Jingle Bells (lyrics)	4/4	9.7 s	200
4		Mary Had a Little Lamb	4/4	11.6 s	160
11	Songs recorded without lyrics	Chim Chim Cheree	3/4	13.5 s	212
12		Take Me Out to the Ballgame	3/4	7.7 s	189
13		Jingle Bells	4/4	9.0 s	200
14		Mary Had a Little Lamb	4/4	12.2 s	160
21	Instrumental pieces	Emperor Waltz	3/4	8.3 s	178
22		Hedwig's Theme (Harry Potter)	3/4	16.0 s	166
23		Imperial March (Star Wars Theme)	4/4	9.2 s	104
24		Eine Kleine Nachtmusik	4/4	6.9 s	140
Mean				10.4 s	176

All stimuli were normalized in volume and kept as similar in length as possible with care taken to ensure that they all contained complete musical phrases starting from the beginning of the piece. The pairs of recordings for the same song with and without lyrics were tempo-matched. The stimuli were presented to the participants in several conditions while EEG was recorded.

1. Stimulus perception with cue clicks
2. Stimulus imagination with cue clicks
3. Stimulus imagination without cue clicks
4. Stimulus imagination without cue clicks, with additional feedback from participants after each trial

Condition 1–3 trials were recorded directly back-to-back. The goal was to lock time and tempo between conditions 1 and 2 through the cue to help identifying overlapping features. Conditions 3 and 4 simulate a more realistic query scenario where the system cannot know the tempo and meter in advance. The presentation was divided into 5 blocks that each comprised all 12 stimuli in randomized order. In total, 60 trials (12 stimuli × 5 blocks) per condition were recorded for each subject.

EEG was recorded from 10 participants (3 male), aged 19–36, with normal hearing and no history of brain injury. A BioSemi Active-Two system was used with 64 + 2 EEG channels sampled at 512 Hz. Horizontal and vertical Electrooculography (EOG) channels were recorded to capture eye movements. The following common-practice pre-processing steps were applied to the raw EEG and EOG data using the MNE-python toolbox by Gramfort et al. (2013) to remove unwanted artifacts. We removed and interpolated bad EEG channels (between 0 and 3 per subject) identified by manual visual inspection. The data was then filtered with a bandpass keeping a frequency range between 0.5 and 30 Hz. This also removed any slow signal drift in the EEG. To remove artifacts caused by eye blinks, we computed independent components using extended Infomax independent component analysis (ICA) as described by Lee et al. (1999) and semi-automatically removed components that had a high correlation with the EOG channels. Afterwards, the 64 EEG channels were reconstructed from the remaining independent components without reducing dimensionality. Furthermore, the data of one participant was excluded for the experiments described in this paper because of a considerable number of trials with movement artifacts due to coughing. Finally, all trial channels were additionally normalized to zero mean and range [−1, 1].

## 3. Related work

Retrieval based on brain wave recordings is still a very young and largely unexplored domain. EEG signals have been used to recognize emotions induced by music perception (Lin et al., 2009; Cabredo et al., 2012) and to distinguish perceived rhythmic stimuli (Stober et al., 2014). It has been shown that oscillatory neural activity in the gamma frequency band (20–60 Hz) is sensitiv to accented tones in a rhythmic sequence (Snyder and Large, 2005) and that oscillations in the beta band (20–30 Hz) increase in anticipation of strong tones in a non-isochronous sequence (Fujioka et al., 2009, 2012; Iversen et al., 2009). While listening to rhythmic sequences, the magnitude of steady state evoked potentials (SSEPs), i.e., reflecting neural oscillations entrained to the stimulus, changes for frequencies related to the metrical structure of the rhythm as a sign of entrainment to beat and meter (Nozaradan et al., 2011, 2012).

EEG studies by Geiser et al. (2009) have further shown that perturbations of the rhythmic pattern lead to distinguishable electrophysiological responses–commonly referred to as Event-Related Potentials (ERPs). This effect appears to be independent of the listener's level of musical proficiency. Furthermore, Vlek et al. (2011) showed that imagined auditory accents imposed on top of a steady metronome click can be recognized from ERPs. However, as usual for ERP analysis to deal with noise in the EEG signal and reduce the impact of unrelated brain activity, this requires averaging the brain responses recorded for many events. In contrast, retrieval scenarios usually only consider single trials. Nevertheless, findings from ERP studies can guide the design of single-trial approaches as demonstrated in subsection 4.1.

EEG has also been successfully used to distinguish perceived melodies. In a study conducted by Schaefer et al. (2011), 10 participants listened to 7 short melody clips with a length between 3.26 and 4.36 s. For single-trial classification, each stimulus was presented for a total of 140 trials in randomized back-to-back sequences of all stimuli. Using quadratically regularized linear logistic-regression classifier with 10-fold cross-validation, they were able to successfully classify the ERPs of single trials. Within subjects, the accuracy varied between 25 and 70%. Applying the same classification scheme across participants, they obtained between 35 and 53% accuracy. In a further analysis, they combined all trials from all subjects and stimuli into a grand average ERP. Using singular-value decomposition, they obtained a fronto-central component that explained 23% of the total signal variance. The related time courses showed significant differences between stimuli that were strong enough for cross-participant classification. Furthermore, a correlation with the stimulus envelopes of up to .48 was observed with the highest value over all stimuli at a time lag of 70–100 ms.

Results from fMRI studies by Herholz et al. (2012) and Halpern et al. (2004) provide strong evidence that perception and imagination of music share common processes in the brain, which is beneficial for training MIIR systems. As Hubbard (2010) concludes in his review of the literature on auditory imagery, “*auditory imagery preserves many structural and temporal properties of auditory stimuli”* and “*involves many of the same brain areas as auditory perception”*. This is also underlined by Schaefer (2011, p. 142) whose “*most important conclusion is that there is a substantial amount of overlap between the two tasks* [music perception and imagination]*, and that ‘internally’ creating a perceptual experience uses functionalities of ‘normal’ perception.”* Thus, brain signals recorded while listening to a music piece could serve as reference data for a retrieval system in order to detect salient elements in the signal that could be expected during imagination as well.

A recent meta-analysis of Schaefer et al. (2013) summarized evidence that EEG is capable of detecting brain activity during the imagination of music. Most notably, encouraging preliminary results for recognizing purely imagined music fragments from EEG recordings were reported in Schaefer et al. (2009) where 4 out of 8 participants produced imagery that was classifiable (in a binary comparison) with an accuracy between 70 and 90% after 11 trials.

Another closely related field of research is the reconstruction of auditory stimuli from EEG recordings. Deng et al. (2013) observed that EEG recorded during listening to natural speech contains traces of the speech amplitude envelope. They used ICA and a source localization technique to enhance the strength of this signal and successfully identify heard sentences. Applying their technique to imagined speech, they reported statistically significant single-sentence classification performance for 2 of 8 subjects with performance increasing when several sentences were combined for a longer trial duration.

More recently, O'Sullivan et al. (2015) proposed a method for decoding attentional selection in a cocktail party environment from single-trial EEG recordings of approximately one minute length. In their experiment, 40 subjects were presented with 2 classic works of fiction at the same time—each one to a different ear—for 30 trials. In order to determine which of the 2 stimuli a subject attended to, they reconstructed both stimuli envelopes from the recorded EEG. To this end, they trained two different decoders per trial using a linear regression approach—one to reconstruct the attended stimulus and the other to reconstruct the unattended one. This resulted in 60 decoders per subject. These decoders where then averaged in a leave-on-out cross-validation scheme. During testing, each decoder would predict the stimulus with the best reconstruction from the EEG using the Pearson correlation of the envelopes as measure of quality. Using subject-specific decoders averaged from 29 training trials, the prediction of the attended stimulus decoder was correct for 89% of the trials whereas the mean accuracy of the unattended stimulus decoder was 78.9%. Alternatively, using a grand-average decoding method that combined the decoders from every other subject and every other trial, they obtained a mean accuracy of 82 and 75% respectively.

## 4. Experiments

Our initial analyses of the OpenMIIR recordings was largely exploratory. Hence, the following subsections cover three very different approaches:

1. ERP-inspired single-trial analysis (subsection 4.1),
2. reconstruction of the audio stimulus envelope from the EEG (subsection 4.2), and
3. extraction of stimulus-related brain activity from the EEG recordings (subsection 4.3).

These approaches increase in complexity, ranging from hand-crafted design to representation learning, i.e., a machine learning pipeline that also includes learning suitable features from the raw EEG data.^2^

The experiments were implemented in Python with the exception of the Matlab code for the tempo estimation experiment described in subsubsection 4.3.5. For neural network training, the framework Theano (Al-Rfou et al., 2016) was used in combination with Blocks and Fuel (van Merriënboer et al., 2015). The code to run the experiments and to generate the plots shown in this paper is made available as open source and linked from the OpenMIIR website. As the OpenMIIR dataset is public domain, this assures full reproducibility of the results presented here.

### 4.1. ERP-inspired single-trial tempo analysis

Our first experiment was inspired by traditional ERP analysis but also incorporated autocorrelation as a common MIR approach to tempo estimation (e.g., Ellis, 2007). This experiment has been described in detail in Sternin et al. (2015). Recordings from 5 participants were used that were available at this point in time. Additionally to the pre-processing steps described in section 2, the EEG recordings were down-sampled to 64 Hz.

#### 4.1.1. Initial ERP-analysis

We started with a basic ERP analysis and focused on the trials recorded for conditions 1–3. Beat annotations were obtain for all beats within the audio stimuli using the dynamic beat tracker described in Ellis (2007) and provided by the *librosa* library.^3^ To this end, the beat tracker was initialized with the known tempo of each stimulus. The quality of the automatic annotations was verified through sonification.

**Figure 1:**
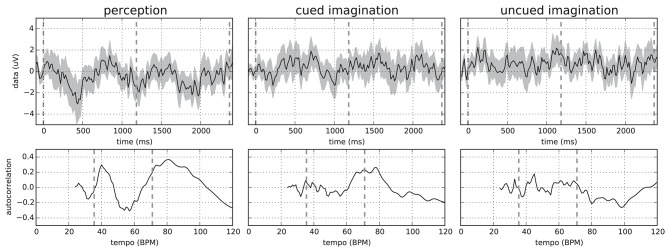
**(Top)** Mean and standard deviation over all 64 EEG channels of the bar-aligned ERPs (without epochs overlap) for “Chim Chim Cheree (lyrics)” in conditions 1–3 for participant P01. Each ERP was averaged over 25 epochs (5 from each trial). **(Bottom)** Corresponding autocorrelation scores in the relevant tempo range. Dashed lines indicate downbeats **(Top)** and the approximate bar tempo of the stimulus plus its lower tempo harmonic **(Bottom)**. Originally published in Sternin et al. (2015).

Given the beat annotations of the stimuli and assuming that the participants would imagine the stimuli at a similar tempo in conditions 2 and 3, we computed bar-aligned ERPs using non-overlapping epochs from 100 ms before to 2.4 s after a downbeat annotation. This length was required to capture slightly more than a single bar for the slowest stimulus–number 23 with a bar length of more than 2.3 s. As expected, the resulting averaged ERPs differed considerably between participants, stimuli, and conditions. Nevertheless, we often observed a periodicity in the averaged signal proportional to the bar length. Figure 1 shows example ERPs for a specific participant and stimulus where this is clearly visible in all conditions.

In order to analyze this periodicity, we computed the autocorrelation curves by comparing each signal with itself at a range of time lags. To this end, we aggregated all 64 EEG channels into a mean signal. We further chose time lags corresponding to the bar tempo range of the stimuli. The lower end of 24 BPM was determined by the choice of the epoch length. Using longer epochs would allow for extending the tempo range to slower tempi, but this would be at the expense of fewer epochs available for averaging.

#### 4.1.2. Limitations and potential pitfalls

In general, more distinct peaks in the autocorrelation were observed in the perception condition. For the two imagination conditions, peaks were more blurred as can also be seen in Figure 1. This is most likely caused by the lack of a time locking mechanism, which allows the imagination tempo to vary—causing bar onsets to deviate from the stimulus-based annotations. This hypothesis is also backed by the observation that artificially jittering the bar onsets results in a decrease in autocorrelation.

The computation of the ERPs benefits from a constant stimulus tempo. The tempo values provided in Table 1 refer to the initial tempo which is also used by the tempo cue. In some stimuli, however, the tempo is not exactly constant but changes slightly over time. In stimulus 22, for instance, the tempo temporarily drops after the first half of the theme at around 8 s. Such deviations further impact the quality of bar-aligned ERPs because of the variable timing within the individual bars.

As a very important detail of the bar-aligned ERP analysis, it is essential to ensure that the bar-aligned epochs do not overlap by rejecting some of the epochs. If they overlap, a single data segment can contribute to multiple epochs at different time points. This can induce misleading autocorrelation peaks that are not supported by the raw data.

#### 4.1.3. Analyzing single trials

Based on the ERP-based observations, the question was whether the tempo could similarly be estimated through autocorrelation from single trials. This posed several challenges. First, there were too few bar-aligned epochs in a single trial to use ERPs. Second, neither the tempo of the stimulus nor the beat annotations should be known a priori in a realistic setting. Therefore, there were no reference points for extracting bar-aligned epochs. Moreover, the problem of possible tempo variance in the imagination conditions needed to be addressed.

**Figure 2:**
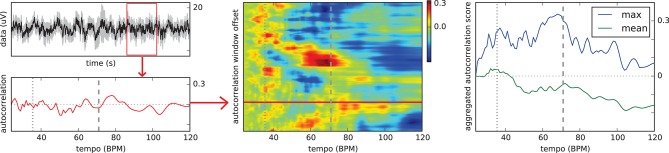
Schema of the proposed tempo estimation technique. All plots refer to the first of the five trials contributing to the imagination ERP of “Chim Chim Cheree (lyrics)” in Figure 1, middle. **Left top:** EEG waveform (mean of all 64 channels) for the whole trial with the red box indicating the sliding window of 2.5 s. **Left bottom:** Autocorrelation curve for this specific segment of the trial. **Middle:** Vertically stacked autocorrelation curves for the whole trial with the red horizontal line indicating the position of the sliding window shown on the left. **Right:** Aggregated autocorrelation scores (mean and max) for the whole trial. Dashed vertical lines indicate the stimulus bar tempo. Dotted vertical lines refer to half the bar tempo. Originally published in Sternin et al. (2015).

Figure 2 illustrates our proposed solution to this problem. A 2.5-seconds sliding window is moved over the mean EEG signal aggregated over all channels. At each position with a hop size of 5 samples at 64 Hz, an autocorrelation curve is computed. The curves for the individual window segments are stacked into a two-dimensional matrix with the first dimension corresponding to the window offset in the trial signal and the second dimension corresponding to the possible tempo values. Hence, each matrix value holds the score for a certain tempo at one specific point in the trial. The scores in the matrix are finally aggregated deriving an estimated tempo value for the trial. While the mean and maximum over all matrix rows often produced significant peaks in the aggregated autocorrelation curve as illustrated in Figure 2, the following heuristic has led to slightly more stable results:

1. In each row, find the pair of tempo values with the maximal combined score.
2. Select the median of all selected pairs.
3. From this pair, return the tempo value with the higher mean value over all rows.

For the evaluation of our approach, we computed the mean absolute error of the estimated tempo and the actual tempo. We also considered the tempo harmonic below and above the correct value, i.e., half or twice the tempo, as a correct result. The prediction error, averaged across all stimuli, varied considerably between participants ranging from 7.07, 7.15, and 8.11 in the three conditions for participant P14 up to 9.81, 10.04, and 12.58 for P12. Furthermore, the results clearly showed a trend that tempo was easier to predict for some stimuli, such as “Chim Chim Cheree” (ID 1 and 11) and “Mary had a little lamb” (ID 4 and 14), than for others. The slowest stimulus, the “Imperial March” (ID 23) had the highest variation of prediction accuracy. These initial results eventually encouraged further research into estimating the stimulus tempo from the EEG using more sophisticated signal processing techniques. This is further described in subsubsection 4.3.5.

### 4.2. Audio stimulus envelope reconstruction

In our second experiment, we attempted to reconstruct the audio stimulus envelopes from the EEG signals, i.e., reversing the black-box signal transformations by the brain and the recording equipment. An approximate reconstruction of the envelopes would be a very useful feature for further retrieval steps such as beat tracking, tempo prediction or stimulus identification. Furthermore, it could also be directly sonified—for instance by shaping a white-noise base signal with the up-sampled envelope. This would be helpful for analysis and for interactive scenarios like brain-computer interfacing where (auditory) feedback is desirable.

**Figure 3:**
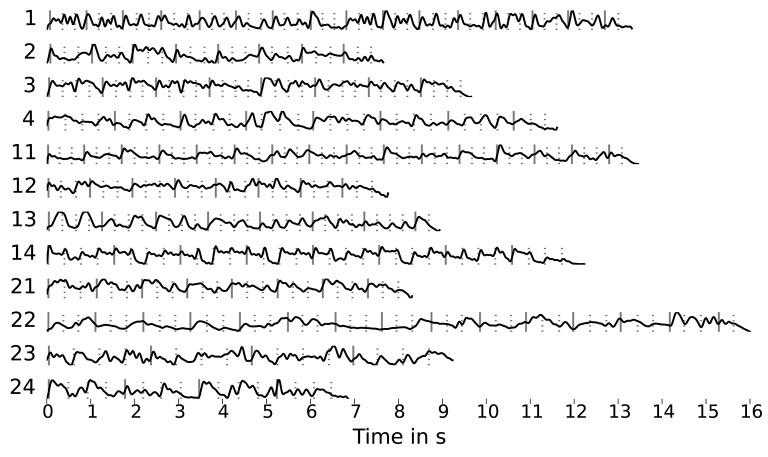
Stimulus envelopes (sampled at 64 Hz, low-pass filtered at 8 Hz) with markers for beats (dashed lines) and downbeats (solid lines) obtained using the dynamic beat tracker by Ellis (2007) as part of the *librosa* library.

Using the method described by O'Sullivan et al. (2015), we attempted to reconstruct and classify the audio envelopes shown in Figure 3. These envelopes were computed by applying the Hilbert transform to the mono audio signal of the stimuli, down-sampling to 64 Hz and low-pass filtering at 8 Hz. The EEG recordings were also down-sampled to 64 Hz matching the envelope sample rate. This rate was chosen to reduce dimensionality and thus limit the number of regression parameters.

The linear reconstruction technique used in O'Sullivan et al. (2015) learns a filter matrix with individual weights for each channel at a range of time lags based on the cross-correlation between the EEG channels and the stimulus envelope. This matrix is then used to convolve the EEG signal to produce the reconstructed stimulus envelope. The size of the matrix and thus the number of parameters to be fit depends on the number of EEG channels and the maximum time lag to be considered.

Directly applying this technique did unfortunately not lead to satisfying results. For the trial-specific decoders, the correlation of the reconstruction and the stimulus envelope was only 0.11 on average with a very high variance of 0.52. Results were also very unstable, i.e., minimally changing the length of the time-lag window generally resulted in very different decoder weights. This eventually produced very poor results when decoder matrices were added together during training, rendering them useless for classification.

We suspect two main reasons for this outcome: Firstly, the trials might be too short for the algorithm to produce stable decoder matrices and secondly, the music envelopes differ significantly from those for speech. We tried to address the second point by using envelopes computed from filtered stimuli versions that emphasized the main voice (using an “inverse-karaoke” filter as described in Duda et al., 2007) and artificial “beat envelopes” derived from the beat and downbeat annotations shown in Figure 3. However, this did not lead to an improvement.

Limiting the maximum time lag to 375 ms and reducing the number of channels through PCA, we were able to reduce the number of parameters and the resulting tendency of over-fitting the filter matrix to the training data. However, the envelope reconstruction quality remained very poor and the resulting (leave-one-out) classification accuracy was not statistically significant. Based on these observations, we concluded that the tested approach which worked well for speech reconstruction is not transferable to our music stimuli. We hypothesize that this is caused by the lack of signal sparsity of the music stimuli.

### 4.3. Extracting music-related brain activity

This experiment aimed to extract brain activity that is related to stimulus perception and imagination using techniques from the field of deep representation learning. Note that this is a much broader focus than the attempted stimulus envelope reconstruction from the previous experiment. Naturally, any EEG signal component correlated with the stimulus envelope would be related to stimulus perception or imagination. But there is potentially much more brain activity that is also related to the music stimuli but not directly helpful for their reconstruction.

The basic pre-processing steps briefly described in section 2 aimed to improve the general signal quality by removing common EEG artifacts. However, there is still the problem that the EEG naturally also records brain activity that is unrelated to music perception or imagination. These signals can be considered as noise with respect to the specific focus of interest. Separating this background noise from the music-related brain activity is a very challenging task. Figuratively speaking, this could be compared to a cocktail-party situation where a listener would like to attend to a specific speaker in a room with many independently ongoing conversations. As an additional complication, the listener is not in the same room as the speakers but in the next room separated by a thick wall—analogously to the EEG equipment that can only measure brain activity from the outside with the skull in between.

This challenge calls for sophisticted signal processing techniques. One newly emerging option is using so-called deep artificial neural networks that over the last decade have become very popular in various application domains such as computer vision, automatic speech recognition, natural language processing, and bioinformatics where they produce state-of-the-art results on various tasks. These networks are able to learn (hierarchies of increasingly) complex features from raw data which is referred to as (deep) representation learning. The learned feature representations can then be used to solve machine learning problems such as a classification tasks. We hypothesized that this approach could also be applied to EEG analysis.

The main problem with applying deep neural networks for EEG analysis is the limited amount of data for training. If all perception trials are clipped to match the length of the shortest stimulus, excluding the cue clicks, the total amount of EEG data recorded for the perception conditions is 63 min from 540 trials. At the same time, each trial has more than 225,000 dimensions at the original sampling rate of 512 Hz. This is very unlike the typical scenarios where deep neural networks are successful.^4^ In such a setting with potentially many network parameters (due to the number of input dimensions) and only a small set of training instances, the neural net is very likely to overfit. I.e., it adapts too much to the training data which results in a poor generalization performance.

We addressed this challenge by focusing on small nets that have few model parameters and by developing a special pre-training technique called similarity-constraint encoding for representation learning. The series of representation learning experiments that eventually led to this technique is described in detail in Stober et al. (2015a). In the following, we summarize the main idea.

#### 4.3.1. Similarity-constraint encoding

The idea of similarity-constraint encoding (SCE) is derived from auto-encoder pre-training (Bengio et al., 2007). An auto-encoder is a neural network that is trained to reconstruct its inputs while its internal representation is limited to make this a non-trivial task—for instance, through a structural bottleneck or regularization of weights or activations. Additionally, the inputs can be corrupted by adding random noise which can result in more robust features (Vincent et al., 2010). This approach has been successfully applied for learning compressed feature representations—usually during an unsupervised pre-training phase—in many domains such as for learning high-level image features (Le, 2013), coding speech spectrograms (Deng et al., 2010) or sentiment analysis (Socher et al., 2011).

EEG data already contain noise from various sources. Furthermore, only a small portion of the recorded brain activity is usually relevant in the context of an experiment. Given only a small dataset, a basic auto-encoder would learn features that represent the full EEG data including noise and irrelevant brain activity. This limits the usefulness of the learned features. For better features, the encoding needs to be more selective. To this end, side information can be used. Demanding that trials belonging to the same class^5^ are encoded similarly facilitates learning features representing brain activity that is stable across trials. Features to be used in classification tasks should furthermore allow for distinguishing between the respective classes. This can be achieved by a training objective that also considers how trials from other classes are encoded.

**Figure 4:**
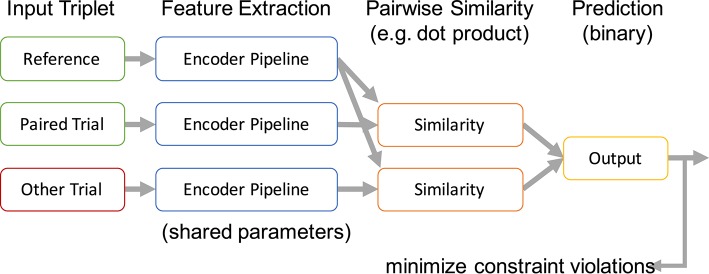
Processing scheme of a similarity-constraint encoder. Originally published in Stober (2017).

In the most basic form, the encoded representations of two trials belonging to the same class are compared with an encoded trial from a different class. The desired outcome of this comparison can be expressed as a *relative similarity constraint* as introduced in Schultz and Joachims (2003). A relative similarity constraint (*a, b, c*) describes a relative comparison of the trials *a*, *b*, and *c* in the form “*a* is more similar to *b* than *a* is to *c*.” Here, *a* is the *reference trial* for the comparison. There exists a vast literature on using such constraints to learn similarity measures in general and for applications within MIR specifically (Lübbers and Jarke, 2009; McFee and Lanckriet, 2010; Stober, 2011; Wolff and Weyde, 2014). Based on this formalization, we define a cost function for learning a feature encoding by combining all pairs of trials (*a, b*) from the same class with all trials *c* belonging to different classes and demanding that *a* and *b* are more similar. The resulting set of trial triplets is then used to train a similarity-constraint encoder network as illustrated in Figure 4.

All trials within a triplet that constitutes a similarity constraint are processed using the same encoder pipeline. This results in three internal feature representations. Based on these, the reference trial is compared with the paired trial and the trial from the other class resulting in two similarity scores. We use the dot product as similarity measure because this matches the way patterns are compared in a neural network classifier and it is also suitable to compare time series. More complex approaches are possible as well, as long as they allow training through backpropagation. The output layer of the similarity constraint encoder finally predicts the trial with the highest similarity score without further applying any additional affine transformations. The whole network can be trained like a common binary classifier, minimizing the error of predicting the wrong trial as belonging to the same class as the reference. The only trainable part is the shared encoder pipeline. This pipeline can be arbitrarily complex—e.g., also include recurrent connections within the pipeline.

After pre-training, the output of the encoder pipeline can be used as feature representation to train a classifier for identifying the actual classes (in contrast to the artificially constructed binary classification problem for pre-training). Alternatively, as we will show later, the features could also be used to train a classifier for different classes than the ones originally used to construct the triplets during pre-training.

#### 4.3.2. Encoder pipeline and classifiers

For all SCE experiments described in the following, the encoder pipeline consisted of a single convolutional layer with a single filter and without a bias term. This filter aggregated the 64 raw EEG channels into a single waveform processing one sample (over all channels) at a time. I.e. it had the shape 64 × 1 (channels × samples) and thus a very small number of parameters. The hyperbolic tangent (tanh) was used as activation function because its output range matched the value range of the network inputs ([−1,1]). No pooling was applied. The number of network and learning hyper parameters was kept as low as possible to minimize their impact.

A linear support vector machine classifier (SVC) was trained using Liblinear (Fan et al., 2008) on

- baseline (1): the raw EEG data,
- baseline (2): the averaged EEG data (mean over all channels as a naïve filter), and
- the output of the pre-trained encoder pipeline.

With this setting, an increase in the stimulus classification accuracy over the baselines can be attributed to a reduction of the signal-to-noise ratio by the encoder pipeline. This could then be interpreted as evidence that the encoder has successfully picked up music-related brain activity.

As additional classifier, a simple neural network (NN) was trained on the encoder pipeline output. This network consisted of a single fully-connected layer with a Softmax non-linearity. No bias term was used. This resulted in one temporal pattern learned for each of the classes, which could then be analyzed. For further comparison, we also trained and end-to-end neural network that had the same structure as the encoder pipeline combined with the neural network classifier but was initialized randomly instead of pre-training. All tested methods are listed in Table 2.

**Table 2:** Accuracies for the three classification tasks: stimulus (12 classes), group (3 classes) and meter (2 classes).

**Classifier**	**Features**	**Classification Accuracy & Significance**		
		**Stimulus (12) (%)**	**Group (3)(%)**	**Meter (2)(%)**
Chance of correct classification for a single trial		8.33	33.33	50.00
Chance accuracy at *p* = 0.001 for 560 trials w.r.t. cumulative binomial distribution		12.22	39.63	56.67
SVC	Raw EEG	18.52 ^***^	40.37 ^**^	62.04 ^**^
SVC	Raw EEG channel mean	12.41 ^****^	38.70 ^***^	58.52 ^****^
End-to-end NN	Raw EEG	18.15 ^***^	37.41 ^****^	60.56 ^***^
Dummy	Output of stimulus classifier		38.89 ^***^	59.63 ^***^
SVC (reference)	Stimulus SCE features	27.59	48.89	69.44
NN	Stimulus SCE features	27.22	48.89	67.78
SVC	Group SCE features		35.37 ^****^	
NN	Group SCE features		34.63 ^****^	
SVC	Meter SCE features			60.19 ^***^
NN	Meter SCE features			58.88 ^****^

*Chance accuracy values are provided for comparison. Significance levels are indicated against the best performing approach (highlighted in red) using McNemar's tests (n = 540, mid-p)*.

** *p < 0.01*,

*** *p < 0.001*,

**** *p < 0.0001*.

#### 4.3.3. Training and evaluation scheme

**Figure 5:**
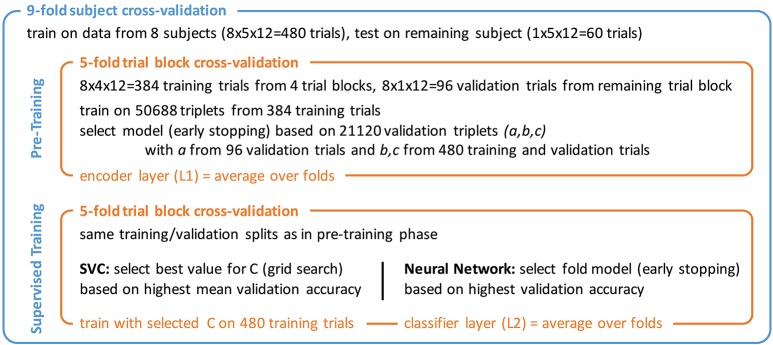
Nested cross-validation scheme with pre-training and supervised training phase. Triplet numbers for SCE pre-training refer to the 12-class stimulus identification task.

A nested cross-validation scheme as shown in Figure 5 was chosen that allowed for using each one of the 540 trials for testing once. The outer 9-fold cross-validation was performed across subjects, training on 8 and testing on the 9th subject. The inner 5-fold cross-validation was used for model selection based on 1 of the 5 trial blocks. Training was divided into two phases.

In the first phase, the encoder pipeline was trained using the proposed similarity-constraint encoding technique with the hinge loss as cost function. Stochastic gradient descent (SGD) with a batch size of 1,000 and the Adam (Kingma and Ba, 2014) step rule was used. Training was stopped after 10 epochs and the model with the lowest binary classification error on the validation triplets was selected. Triplets were constructed such that all trials within a triplet belonged to the same subject as the simple encoder pipeline likely could not easily compensate inter-subject differences. The validation triplets consisted of a reference trial from the validation trials and the other two trials drawn from the combined training and validation set of the inner cross-validation. This way, a reasonable number of validation triplets could be generated without sacrificing too many trials for validation.^6^ The final encoder filter weights were computed as mean of the 5-fold models. The output of this filter was used to compute the features for the second training phase.

In the second phase, the two classifiers were trained. For the SVC, the optimal value for the parameter C that controls the trade-off between the model complexity and the proportion of non-separable training instance was determined through a grid search during the inner cross-validation. For the neural network classifier, 5 fold models were trained for 100 epochs using SGD with batch size 120, the Adam step rule, and the hinge loss as cost function. The best models were selected based on the classification performance on the validation trials and then averaged to obtain the final classifier.

#### 4.3.4. Stimulus identification

**Figure 6:**
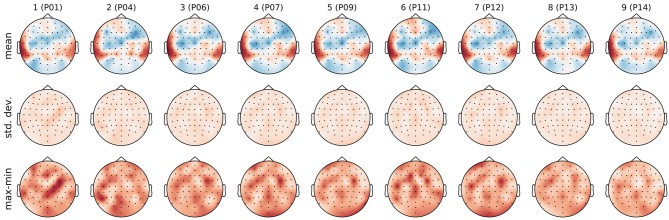
Topographic visualization of the learned filter weights aggregated over the 5 inner cross-validation folds within each outer cross-validation fold. **(Top)** mean. **(Middle)** standard deviation. **(Bottom)** range (maximum–minimum). Columns correspond to outer cross-validation folds with the id of the test subject as column label. The mean filters in the top row were used to compute the features for the supervised training phase. All plots use the same color map and range.

As first classification task, we investigated stimulus identification. There are 12 perfectly balanced classes–one for each stimulus. Figure 6 shows the filters learned in the pre-training phase of each outer cross-validation fold as well as the standard deviation and ranges for the weights within the 5 inner cross-validation folds. The filter weights only differed in small details across folds. However, sometimes the polarity of the weights had flipped. To avoid cancellation effects during aggregation, the polarity was normalized based on the sign of the weight for channel T7 (next to the left ear), which always had a high absolute value.

**Figure 7:**
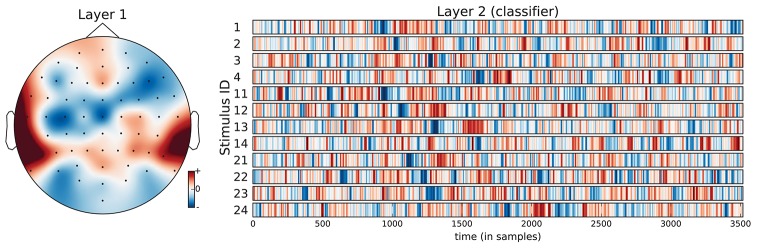
Visualization of the average neural network parameters (from the 9 outer cross-validation folds) for stimulus classification. Layer 1: mean of convolutional layers from the pre-trained encoders (SCE), i.e., mean over the top row in Figure 6. The filter weights only differed in small details across folds. Layer 2: mean of classifier layers trained in the supervised phase.

The magnitude of the channel weights in the pre-trained filters (which are further aggregated in Figure 7) indicates how much the respective EEG channels are contributing to the aggregated signal. The electrodes within the dark red areas that appear bilaterally towards the back of the head lie directly over the auditory cortex. These electrodes may be picking up on brain activation from the auditory cortex that is modulated by the perception of the stimuli. The electrodes within the blue areas that appear more centrally may be picking up on the cognitive processes that occur as a result of the brain processing the music at a higher level.

**Figure 8:**
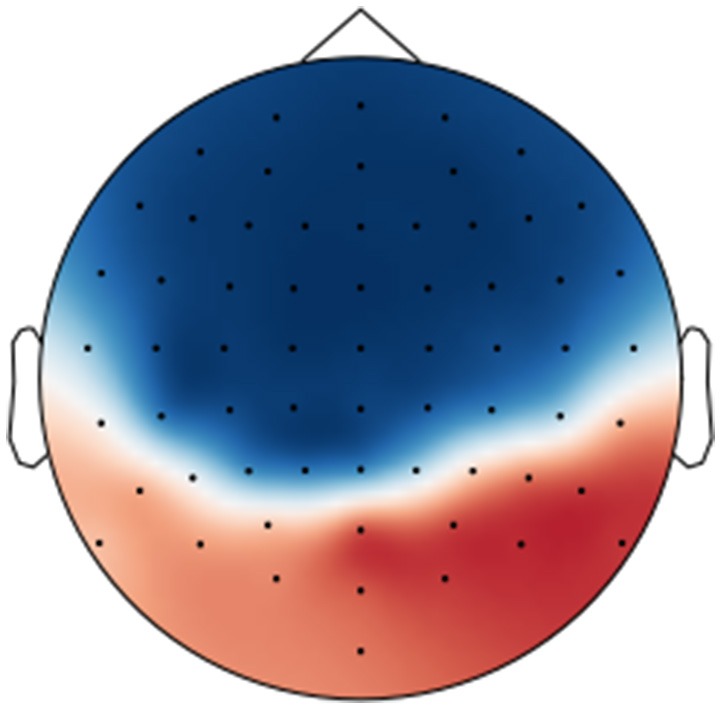
Visualization of the forward model (deconvolutional filter) trained to reconstruct the originally recorded EEG signals from the encoder output over all perception trials.

However, as pointed out by Haufe et al. (2014), model parameters for classification or decoding should not be directly interpreted in terms of the brain activity as they depend on all noise components in the data, too. Instead, a forward model should be derived that explains how the measured signals were generated from the neural sources. We applied the proposed regression approach and trained a deconvolutional filter that reconstructs the original EEG signal from the encoder output by minimizing the mean squared error between the reconstructed and the actual signal over all trials. For each trial, we used the encoder from the respective outer cross-validation fold. The resulting deconvolutional filter is shown in Figure 8.

Table 2 (column “stimulus”) lists the classification accuracy for the tested approaches. Remarkably, all values were significantly above chance. Even for baseline 2, the value of 12.41% was significant at *p* = 0.001. This significance value was determined by using the cumulative binomial distribution to estimate the likelihood of observing a given classification rate by chance. To evaluate whether the differences in the classification accuracies produced by the different methods are statistically significant, McNemar's tests using the “mid-p” variant suggested in Fagerland et al. (2013) were applied. The obtained significance levels are indicated in Table 2 for a comparison with the best performing approach—using SVC in combination with the SCE features learned for stimulus identification. The very significant improvement of the classification accuracy over the two baselines and the neural network trained end-to-end is a strong indicator for a reduction of the signal-to-noise ratio. Notably, the pre-trained filter is very superior to the naïve filter of baseline 2 that was actually harmful judging from the drop in accuracy.

**Figure 9:**
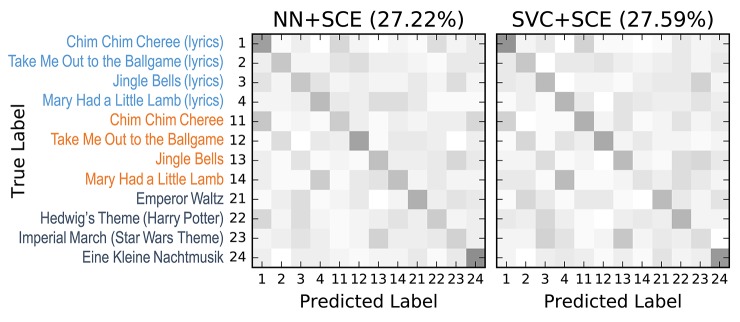
12-class confusion matrices for the music stimuli (listed on the left) for the classifiers trained on the stimulus SCE output. Middle: SVC. Right: Neural network classifier (NN). Results were aggregated from the 9 outer cross-validation folds (*n* = 540). Originally published in Stober (2017).

**Figure 10:**
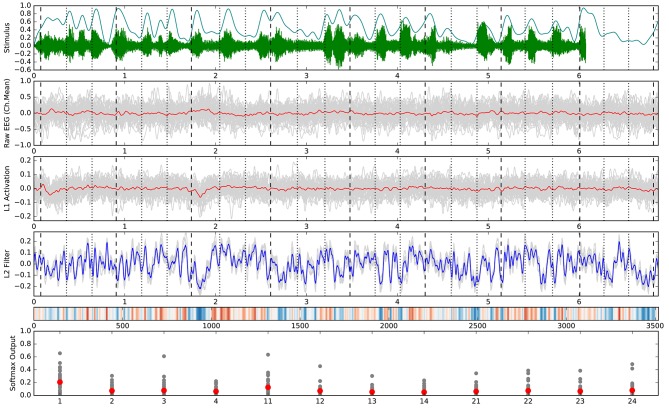
Detailed analysis of all trials belonging to stimulus 1. Vertical marker lines indicate beats (dotted) and downbeats (dashed). The horizontal axis in rows 1–5 corresponds with the time in seconds or samples (row 5). **(Top)** Audio stimulus (green) and envelope (cyan). **(2nd row)** Raw EEG averaged over all 64 channels per trial (gray) and overall mean (red). This is identical to the SVC input for baseline 2. **(3rd row)** Encoder output (activation) for the individual trials (gray) and overall mean (red). **(4th row)** Patterns learned by the neural network classifier for this class in the 9 folds of the outer cross-validation (gray) and overall mean (blue). **(5th row)** Alternative visualization (as in Figure 7) of the averaged pattern from row 4. **(Bottom)** Softmax output of the neural network classifier for the individual trials (gray) and overall mean (red) with class labels on the horizontal axis. All outputs were generated using the respective test trials for each fold model in the outer cross-validation.

The confusion matrices for the classifiers trained on the encoder output are shown in Figure 9. Apart from the main diagonal, two parallel diagonals can be seen that indicate confusion between stimuli 1–4 and their corresponding stimuli 11–14, which are tempo-matched recordings of songs 1–4 without lyrics. Analyzing the averaged neural network parameters visualized in Figure 7 shows similar temporal patterns for these stimuli pairs.^7^ A detailed analysis of the network layer activations as shown in Figure 10 reveals noticeable peaks in the encoder output and matching weights with high magnitude in the classifier layer that often coincide with downbeats–i.e., the first beat within each measure, usually with special musical emphasis. These peaks are not visible in the channel-averaged EEG (baseline 2). Thus, it can be concluded that the encoder filter has successfully extracted a component from the EEG signal that contains musically meaningful information.

Both, the systematic confusion of stimuli 1–4 with their corresponding tempo-matched versions without lyrics (stimuli 11–14) as well as the temporal patterns learned by the neural network classifier are strong indicators against a possible “horse” classifier. Sturm (2014) defines a “horse” as “a system appearing capable of a remarkable human feat […] but actually working by using irrelevant characteristics (confounds).” In this specific context, a “horse” might base the classification on signal components unrelated to music cognition. An additional behavioral experiment where 8 subjects judged the similarity of each stimulus pair confirmed the parallel diagonals observed in the confusion matrices. Measuring the time required to recognize the individual music stimuli yielded average values of 1–3 s that did not correlate with the third-downbeat peaks in the temporal patterns of the classifier. This suggests that the peak is not related to brain activity caused by stimulus recognition but rather by musical features of the stimuli.

#### 4.3.5. Tempo estimation revisited

In a follow-up experiment published in Stober et al. (2016) that also picks up the thread from our tempo analysis experiment described in subsection 4.1, we used the stimulus SCE features as input to a sophisticated tempo estimation technique provided by the Tempogram Toolbox.^8^ This technique has been originally developed for analyzing audio recordings. To compute a tempogram, a given music audio signal is first transformed into a novelty curve that captures note onset information—for instance, as the positive part of a spectral flux as described in Grosche and Müller (2011a). Through a short-time Fourier analysis of the novelty curve, the audio tempogram is derived that reveals how dominant different tempi are at a given time point in the audio signal. Aggregating a tempo histogram along the time axis yields a tempo histogram where peaks indicate the predominant tempo within the piece.

We applied the same processing pipeline for the perception EEG data of participants P09 to P14 by directly interpreting the EEG signal filtered by the SCE encoder pipeline as novelty curve. We were able to observe peaks in the derived tempo histograms that sometimes highly correlated with the stimulus tempo. Averaging tempogram histograms over trials and participants overall stabilized the tempo estimation. Remarkably, results seemed to strongly depend on the music stimuli. For the first 8 stimuli (1–4 and 11–14), i.e., the songs recorded with and without lyrics, the tempo extraction seemed to work better than for the last 4 (21–24), i.e., the instrumental pieces. Exploring this effect was beyond the scope of this small study. To uncover and properly understand the underlying factors, a large-scale music perception experiment using stimuli with systematically adapted tempi would be needed. Possible reasons might be the complexity of the music stimuli, the presence of lyrics, the participants, or the applied methodology and techniques. Investigating these issues could be a starting point for interdisciplinary research between MIR and music cognition.

#### 4.3.6. Group classification

As described in section 2 and shown in Table 1, the 12 music stimuli can be grouped into 3 groups of 4 stimuli each: songs recorded with lyrics (stimuli 1–4), songs recorded without lyrics (stimuli 11–14), and instrumental pieces (stimuli 21–24). Using these three perfectly balanced classes, 184,320 training triplets and 72,960 validation triplets were available for each inner cross-validation fold during SCE pre-training. Here, the SCE pre-training did not result in a suitable feature representation as indicated by the inferior classification accuracy compared to the baselines shown in Table 2 (column “groups”). As a likely reason, the SCE learning problem may me ill-posed, i.e., the encoder pipeline may not have been sufficiently complex to learn a transformation of the raw EEG that makes trials within groups more similar to each other than to trials from the other groups.

**Figure 11:**
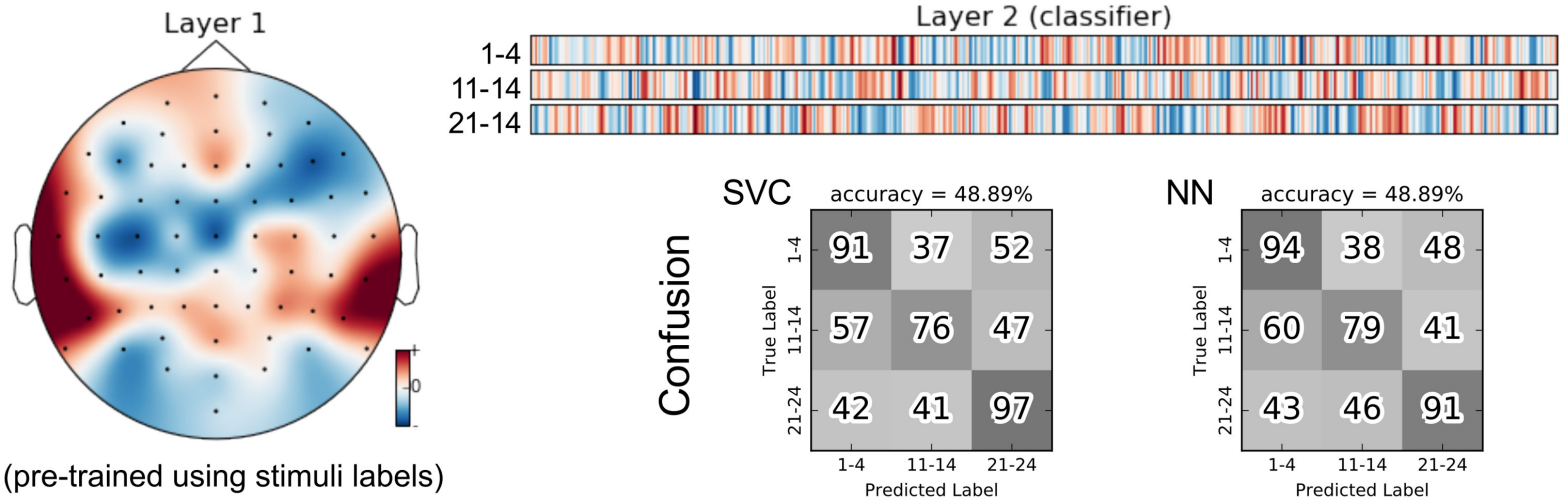
Visualization of the average neural network parameters (from the 9 outer cross-validation folds) for group classification (stimuli 1–4: songs recorded with lyrics, stimuli 11–14: songs recorded without lyrics, stimuli 21–24: instrumental pieces). Layer 1: mean of convolutional layers from the pre-trained encoders (SCE) using the stimuli labels. Layer 2: mean of classifier layers trained in the supervised phase.

As an alternative, we trained the group classifiers on the feature representation from the stimulus classification task. This resulted in a substantial increase in classification accuracy of roughly 10%. We further added a “dummy” baseline classifier that just derived the group class labels from the predicted stimulus labels. The difference in accuracy indicates that the stimulus SCE features seem to capture some relevant information for the group classification task beyond what is necessary to recognize the stimuli. Similarly to Figures 7 and 9 for the stimulus classification task, Figure 11 shows the parameters of the neural network classifier averaged over the 9 outer cross-validation folds as well as the confusion matrices for the two group classifiers trained on the stimulus SCE features. The temporal patterns learned by the classifier are currently subject of further analysis.

#### 4.3.7. Meter classification

**Figure 12:**
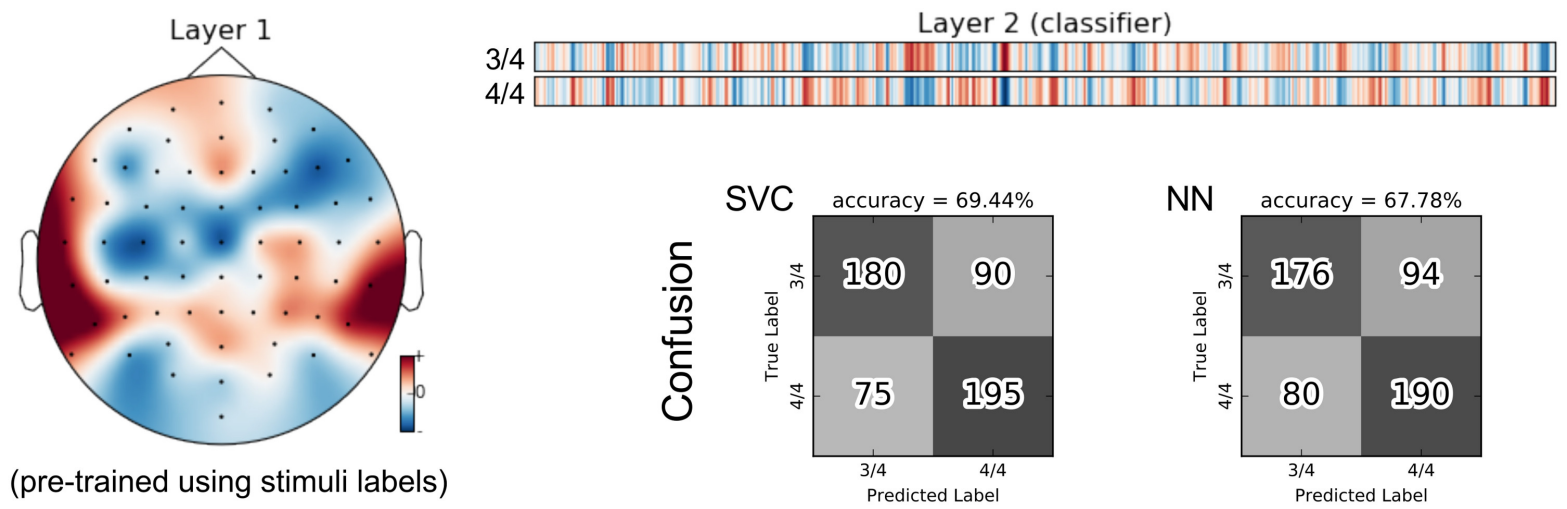
Visualization of the average neural network parameters (from the 9 outer cross-validation folds) for meter classification. Layer 1: mean of convolutional layers from the pre-trained encoders (SCE) using the stimuli labels. Layer 2: mean of classifier layers trained in the supervised phase.

There are two perfectly balanced classes with respect to meter as half of the stimuli are in 3/4 meter and the others are in 4/4 meter. With these class labels, 211,968 training triplets and 83,520 validation triplets are available for each inner cross-validation fold during SCE pre-training. As for the group classification, the resulting feature representation is not helpful for this classification task. Instead, using the stimulus SCE features again results in the best performance that is roughly 9% higher. Figure 12 shows the parameters of the neural network classifier averaged over the 9 outer cross-validation folds as well as the confusion matrices for the two group classifiers trained on the stimulus SCE features.

The inferior performance of the meter SCE features may again be attributed to complexity limitations of the simple convolutional encoder pipeline. We are currently investigating more complex encoders that also incorporate recurrent components to capture temporal patterns within the encoder already.

#### 4.3.8. Classifying EEG from the imagination conditions

All SCE-based experiments described above focused on perception data. Applying the same pre-training technique to the data from the imagination conditions has so far not led to significant classification results or to the discovery of meaningful or interesting patterns. Also, using the encoder trained on the perception data to filter the imagination trials before training the classifier was not successful. As possible reason for this, we suspect—at least for the current encoder design—that timing and synchronization in the imagination trials are insufficiently accurate. This makes it hard to learn an encoder that produced similar temporal patterns or—given a successfully pre-trained encoder—to learn temporal patterns for classification that generalize well. Different encoder designs that can compensate temporal variance may lead to better results. This needs to be further investigated. However, focusing on the perception data for now in order to improve the analysis methods appears to be more promising.

## 5. Discussion

### 5.1. Proposal of an MIR-driven research approach

**Figure 13:**
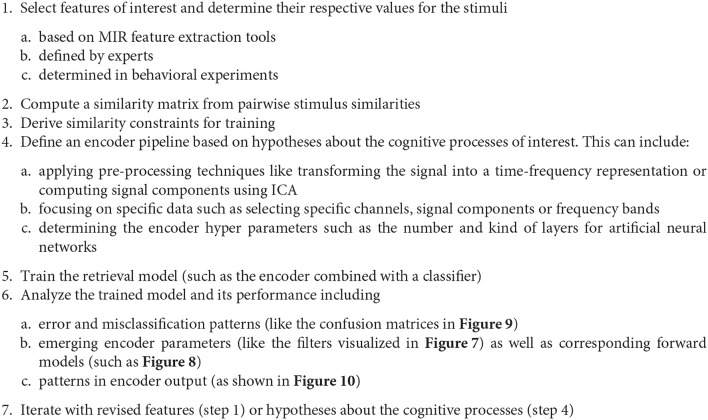
Outline of the proposed MIR-driven research approach using similarity-constraint encoding as a specific example.

Based on the findings from our representation learning experiments described in subsection 4.3, we can derive the following general MIR-driven approach to analyzing music perception and imagination data as outlined in Figure 13. We start by choosing a specific music feature—that necessarily has to be present in the respective music stimuli—and attempt to retrieve it from the recorded brain signals. Representation learning techniques like similarity-constraint encoding allow for finding signal filters that extract relevant components from the recorded brain signals given that we have chosen a suitable encoder pipeline. This choice should be hypothesis-driven and informed by findings from cognitive neuroscience. If the trained encoder pipeline indeed improves the signal-to-noise ratio and consequently the retrieval performance, this can be seen as supporting evidence for the hypothesis that guided the encoder design. Analyzing the emerging network parameters and activation patterns might further allow for learning more about the underlying cognitive processes. Failure could be attributed to poor encoder design choices and question the underlying hypothesis, or it could be caused by limitations of the dataset. (For instance, there might be a bias within the dataset caused by the choice of the stimuli or the participants.) The impact of the latter should naturally be minimized through the study design.

### 5.2. Interpretation of temporal classifier and activation patterns

The neural networks trained so far seem still simple enough to allow for interpretation of the learned parameters by domain experts and facilitate findings about the cognitive processes. Most remarkably, the temporal patterns learned by the neural network classifier for the stimulus identification task show prominent signal peaks at the third downbeat (i.e., the beginning of the third bar) for almost all stimuli. They can be clearly recognized in the visualization of the averaged model parameters in Figure 7. There are also noticeable matching peaks in the encoder filter activation as shown in Figure 10 for one of the stimuli. This raises the question which cognitive process could explain these patterns and calls for further investigation by domain experts from music cognition.

### 5.3. Lessons learned from the OpenMIIR study design

In the way it has been used so far, similarity-constraint encoding imposes a strong regularization assumption that requires a very tight synchronization of the trials to identify good filter parameters. This is problematic if synchronization between the stimuli and the recorded EEG signals is poor like in the imagination conditions. Different encoder designs–for instance including temporal pooling operations–might be able to compensate the lack of tight synchronizations. But generally, it seems very desirable for representation learning to ensure synchronization by experimental design in the first place. In the design of our study to collect the first OpenMIIR dataset, we decided against having a metronome click for synchronization during imagination trials in order to avoid artifacts caused by the audio stimulation. It seems now like the downside of having such artifacts is outweighed by the possible benefits of tightly controlling the imagination tempo. Of course, the added metronome clicks in the background would have to be exactly identical in tempo, loudness etc. for all stimuli. Otherwise, they would easily allow for distinguishing the stimuli by a “horse” classifier.^9^ Hence, all stimuli would need to be in the same tempo (or multiples) as the click.

Another issue is the variable length of the stimuli caused by using full musical phrases in the original study. We ended up cutting all trials to the length of the shortest one for our representation learning approach. Zero-padding the shorter trials instead would have easily given away their identity leading to useless feature representations. To avoid recording likely unused EEG data, it seems more desirable to have equal-length trials–even as this means to stop in the middle of a musical phrase. Having tempo-synced stimuli makes finding good cut points already easier.

Furthermore, switching to imagination trials with a metronome click would also rule out conditions 3 and 4 as listed in section 2. With half as many conditions, already twice as many trials could be recorded in the same time. Additionally reducing the number of stimuli and the stimuli length would allow for further increasing the number of trials per class and condition. This could allow us to collect enough data for learning within-subject feature representations and retrieval models. Based on these considerations, we are currently designing a follow-up OpenMIIR study to collect another EEG dataset.

## 6. Conclusions and outlook

Less than four years have passed since the subject of MIIR was first discussed during the “Unconference” (Anglade et al., 2013) at the International Society of Music Information Retrieval Conference (ISMIR) in 2012. ISMIR 2016 already featured a well-attended tutorial on the “Introduction to EEG Decoding for Music Information Retrieval Research” and for the first time, the annual seminar on Cognitively based Music Informatics Research (CogMIR) was co-located as a satellite event which drew the attention of many main-conference attendees. This is evidence for the increasing interest within the MIR community to combine MIR and music cognition research.

The goal of the OpenMIIR initiative is to foster interdisciplinary exchange and collaborations between these two fields. To this end, we introduced the OpenMIIR dataset in 2015—an public-domain EEG dataset intended to enable MIR researchers to venture into the domain of music imagery and develop novel methods without the need for special EEG equipment. This paper summarized our findings from a first series of largely exploratory experiments addressing several MIIR tasks with this dataset. For some tasks—especially when working with data from the imagination conditions—our approaches failed or did not perform as expected. We have hypothesized why this might be the case and derived ideas for a follow-up EEG study to collect a second dataset.

A first success of our efforts is our proposed similarity-constraint encoding approach for extracting music-related brain activity of EEG recordings. Using this technique, we were able to train simple spatial filters that significantly improve the signal-to-noise ratio for the perception data in several classification tasks. There is a lot of potential for improving the classification accuracy by using more complex encoders that possibly comprise multiple layers of neurons and recurrent connections. Investigating such options is one major direction of our ongoing research efforts. We have also obtained encouraging first results by applying MIR techniques from the Tempogram Toolbox for estimating the stimulus tempo from the perception EEG recordings. This experiment nicely showcases how well-established MIR techniques for music audio analysis can also be applied to music cognition data.

We hope that our work described here inspires other MIR researchers to try their methods in this emerging interdisciplinary field and encourages music cognition researchers to share their datasets and engage in an exchange with the MIR community. Everybody interested is invited to contribute and collaborate within the OpenMIIR initiative. Further information about the OpenMIIR initiative can be found at https://openmiir.github.io where apart from the OpenMIIR dataset itself, the code to run the described experiments is shared and constantly being updated.

## Author contributions

The author confirms being the sole contributor of this article and approved it for publication.

### Conflict of interest statement

The author declares that the research was conducted in the absence of any commercial or financial relationships that could be construed as a potential conflict of interest.

## Footnotes

^1^The dataset is available at https://github.com/sstober/openmiir

^2^An introduction and overview of representation learning is, for instance, provided by Bengio et al. (2013).

^3^https://github.com/bmcfee/librosa

^4^For comparison, a 224-by-224 RGB image in the Imagenet dataset has roughly 150,000 dimensions—about two-thirds of the size of the EEG trials. However, Imagenet contains millions of labeled images for training.

^5^There are several ways to assign the trials to classes based on the stimulus meta-data such as the stimulus id, the meter, or the presence or absence of lyrics.

^6^At least 2 of the 5 trials per class and subject are required to construct within-subject triplets.

^7^The average model is only for illustration and analysis. For testing, the respective outer cross-validation fold model was used for each trial.

^8^The Tempogram Toolbox contains MATLAB implementations for extracting various types of tempo and pulse related audio representations (Grosche and Müller, 2011b) A free implementation can be obtained at https://www.audiolabs-erlangen.de/resources/MIR/tempogramtoolbox.

^9^Cf. Section 4.3.4 for a discussion of the “horse” phenomenon.

## References

[B1] Al-Rfou R. Alain G. Almahairi A. Angermueller C. Bahdanau D. Ballas N. (2016). Theano: a python framework for fast computation of mathematical expressions. arXiv:1605.02688.

[B2] Anglade A. Humphrey E. Schmidt E. Stober S. Sordo M. (2013). Demos and late-breaking session of the thirteenth international society for music information retrieval conference (ismir 2012). Comput. Music J. 37, 91–93. 10.1162/COMJ_r_00171

[B3] Bengio Y. Courville A. Vincent P. (2013). Representation learning: a review and new perspectives. Patt. Anal. Mach. Intell. IEEE Trans. 35, 1798–1828. 10.1109/TPAMI.2013.50 23787338

[B4] Bengio Y. Lamblin P. Popovici D. Larochelle H. (2007). Greedy layer-wise training of deep networks, in Advances in Neural Information Processing Systems 19. 18254699

[B5] Cabredo R. Legaspi R. S. Inventado P. S. Numao M. (2012). An emotion model for music using brain waves, in Proceedings of the 13th International Society for Music Information Retrieval Conference, ISMIR 2012, Mosteiro S. Bento Da Vitória, eds Gouyon F. Herrera P. Martins L. G. Müller M. (Porto), 265–270.

[B6] Cowen A. S. Chun M. M. Kuhl B. A. (2014). Neural portraits of perception: reconstructing face images from evoked brain activity. Neuroimage 94, 12–22. 10.1016/j.neuroimage.2014.03.018 24650597 PMC4028096

[B7] Deng L. Seltzer M. L. Yu D. Acero A. Mohamed A.-R. Hinton G. E. (2010). Binary Coding of Speech Spectrograms Using a Deep Auto-Encoder. Interspeech.

[B8] Deng S. Srinivasan R. D'Zmura M. (2013). Cortical Signatures of Heard and Imagined Speech Envelopes. Technical report, DTIC.

[B9] Duda A. Nürnberger A. Stober S. (2007). Towards query by singing/humming on audio databases, in Proceedings of the 8th International Conference on Music Information Retrieval, ISMIR 2007, eds Dixon S. Bainbridge D. Typke R. (Vienna), 331–334.

[B10] Ellis D. P. W. (2007). Beat tracking by dynamic programming. J. New Music Res. 36, 51–60. 10.1080/09298210701653344

[B11] Fagerland M. Lydersen S. Laake P. (2013). The mcnemar test for binary matched-pairs data: mid-p and asymptotic are better than exact conditional. BMC Med. Res. Methodol. 13:1. 10.1186/1471-2288-13-91 23848987 PMC3716987

[B12] Fan R.-E. Chang K.-W. Hsieh C.-J. Wang X.-R. Lin C.-J. (2008). Liblinear: a library for large linear classification. J. Mach. Learn. Res. 9, 1871–1874.

[B13] Fujioka T. Trainor L. J. Large E. W. Ross B. (2009). Beta and gamma rhythms in human auditory cortex during musical beat processing. Anna. N.Y. Acad. Sci. 1169, 89–92. 10.1111/j.1749-6632.2009.04779.x 19673759

[B14] Fujioka T. Trainor L. J. Large E. W. Ross B. (2012). Internalized timing of isochronous sounds is represented in neuromagnetic beta oscillations. J. Neurosci. 32, 1791–1802. 10.1523/JNEUROSCI.4107-11.2012 22302818 PMC6703342

[B15] Geiser E. Ziegler E. Jancke L. Meyer M. (2009). Early electrophysiological correlates of meter and rhythm processing in music perception. Cortex 45, 93–102. 10.1016/j.cortex.2007.09.010 19100973

[B16] Gramfort A. Luessi M. Larson E. Engemann D. Strohmeier D. Brodbeck C.. (2013). MEG and EEG data analysis with MNE-Python. Front. Neurosci. 7:267. 10.3389/fnins.2013.00267 24431986 PMC3872725

[B17] Grosche P. Müller M. (2011a). Extracting predominant local pulse information from music recordings. IEEE Trans. Audio Speech Lang. Process. 19, 1688–1701. 10.1109/TASL.2010.2096216

[B18] Grosche P. Müller M. (2011b). Tempogram toolbox: MATLAB implementations for tempo and pulse analysis of music recordings, in Late-Breaking News of the International Society for Music Information Retrieval Conference (ISMIR) (Miami, FL).

[B19] Halpern A. R. Zatorre R. J. Bouffard M. Johnson J. A. (2004). Behavioral and neural correlates of perceived and imagined musical timbre. Neuropsychologia 42, 1281–1292. 10.1016/j.neuropsychologia.2003.12.017 15178179

[B20] Haufe S. Meinecke F. Görgen K. Dähne S. Haynes J.-D. Blankertz B.. (2014). On the interpretation of weight vectors of linear models in multivariate neuroimaging. Neuroimage 87, 96–110. 10.1016/j.neuroimage.2013.10.067 24239590

[B21] Herholz S. Halpern A. Zatorre R. (2012). Neuronal correlates of perception, imagery, and memory for familiar tunes. J. Cogn. Neurosci. 24, 1382–1397. 10.1162/jocn_a_00216 22360595

[B22] Horikawa T. Tamaki M. Miyawaki Y. Kamitani Y. (2013). Neural decoding of visual imagery during sleep. Science 340, 639–642. 10.1126/science.1234330 23558170

[B23] Hubbard T. L. (2010). Auditory imagery: empirical findings. Psychol. Bull. 136, 302–329. 10.1037/a0018436 20192565

[B24] Iversen J. R. Repp B. H. Patel A. D. (2009). Top-down control of rhythm perception modulates early auditory responses. Anna. N.Y. Acad. Sci. 1169, 58–73. 10.1111/j.1749-6632.2009.04579.x 19673755

[B25] Kaneshiro B. Dmochowski J. P. (2015). Neuroimaging methods for music information retrieval: current findings and future prospects, in Proceedings of the 16th International Society for Music Information Retrieval Conference (ISMIR'15), 538–544.

[B26] Kingma D. Ba J. (2014). Adam: a method for stochastic optimization. arXiv:1412.6980.

[B27] Le Q. (2013). Building high-level features using large scale unsupervised learning, in 2013 IEEE International Conference on Acoustics, Speech and Signal Processing (ICASSP), 8595–8598.

[B28] Lee T. Girolami M. Sejnowski T. J. (1999). Independent component analysis using an extended infomax algorithm for mixed subgaussian and supergaussian sources. Neural Comput. 11, 417–441. 10.1162/089976699300016719 9950738

[B29] Lin Y.-P. Jung T.-P. Chen J.-H. (2009). EEG dynamics during music appreciation, in Annual International Conference of the IEEE Engineering in Medicine and Biology Society, 2009. EMBC 2009 (Minneapolis, MN: IEEE), 5316–5319. 10.1109/IEMBS.2009.5333524 19964121

[B30] Lübbers D. Jarke M. (2009). Adaptive multimodal exploration of music collections, in Proceedings of the 10th International Society for Music Information Retrieval Conference (ISMIR'09), 195–200.

[B31] McFee B. Lanckriet G. R. G. (2010). Metric learning to rank, in Proceedings of the 27th International Conference on Machine Learning (ICML'10) (Haifa), 775–782.

[B32] Miranda E. R. Castet J. (eds.). (2014). Guide to Brain-Computer Music Interfacing. London: Springer.

[B33] Miyawaki Y. Uchida H. Yamashita O. Sato M.-A. Morito Y. Tanabe H. C.. (2008). Visual image reconstruction from human brain activity using a combination of multiscale local image decoders. Neuron 60, 915–929. 10.1016/j.neuron.2008.11.004 19081384

[B34] Nishimoto S. Vu A. Naselaris T. Benjamini Y. Yu B. Gallant J. (2011). Reconstructing visual experiences from brain activity evoked by natural movies. Curr. Biol 21, 1641–1646. 10.1016/j.cub.2011.08.031 21945275 PMC3326357

[B35] Nozaradan S. Peretz I. Missal M. Mouraux A. (2011). Tagging the neuronal entrainment to beat and meter. J. Neurosci. Off. J. Soc. Neurosci. 31, 10234–10240. 10.1523/JNEUROSCI.0411-11.2011 21753000 PMC6623069

[B36] Nozaradan S. Peretz I. Mouraux A. (2012). Selective neuronal entrainment to the beat and meter embedded in a musical rhythm. J. Neurosci. 32, 17572–17581. 10.1523/JNEUROSCI.3203-12.2012 23223281 PMC6621650

[B37] O'Sullivan J. A. Power A. J. Mesgarani N. Rajaram S. Foxe J. J. Shinn-Cunningham B. G.. (2015). Attentional selection in a cocktail party environment can be decoded from single-trial EEG. Cereb. Cortex 25, 1697–1706. 10.1093/cercor/bht355 24429136 PMC4481604

[B38] Schaefer R. S. (2011). Measuring the Mind's Ear EEG of Music Imagery. PhD thesis, S.l., Nijmegen.

[B39] Schaefer R. S. Blokland Y. Farquhar J. Desain P. (2009). Single trial classification of perceived and imagined music from EEG, in Proceedings of the 2009 Berlin BCI Workshop (Berlin).

[B40] Schaefer R. S. Desain P. Farquhar J. (2013). Shared processing of perception and imagery of music in decomposed EEG. Neuroimage 70, 317–326. 10.1016/j.neuroimage.2012.12.064 23298753

[B41] Schaefer R. S. Farquhar J. Blokland Y. Sadakata M. Desain P. (2011). Name that tune: decoding music from the listening brain. Neuroimage 56, 843–849. 10.1016/j.neuroimage.2010.05.084 20541612

[B42] Schultz M. Joachims T. (2003). Learning a distance metric from relative comparisons, in Advances in Neural Information Processing Systems, 41–48.

[B43] Snyder J. S. Large E. W. (2005). Gamma-band activity reflects the metric structure of rhythmic tone sequences. Cogn. Brain Res. 24, 117–126. 15922164 10.1016/j.cogbrainres.2004.12.014

[B44] Socher R. Pennington J. Huang E. H. Ng A. Y. Manning C. D. (2011). Semi-supervised recursive autoencoders for predicting sentiment distributions, in Proceedings of the Conference on Empirical Methods in Natural Language Processing (Association for Computational Linguistics), 151–161.

[B45] Sternin A. Stober S. Grahn J. A. Owen A. M. (2015). Tempo estimation from the eeg signal during perception and imagination of music, in 1st International Workshop on Brain-Computer Music Interfacing/11th International Symposium on Computer Music Multidisciplinary Research (BCMI/CMMR'15) (Plymouth).

[B46] Stober S. (2011). Adaptive distance measures for exploration and structuring of music collections, in Proceedings of AES 42nd Conference on Semantic Audio (Ilmenau), 275–284.

[B47] Stober S. (2017). Learning discriminative features from electroencephalography recordings by encoding similarity constraints, in Proceedings of 42nd IEEE International Conference on Acoustics, Speech and Signal Processing (ICASSP′*17)* (New Orleans, LA), 6175–6179. 10.1109/ICASSP.2017.7953343

[B48] Stober S. Cameron D. J. Grahn J. A. (2014). Using convolutional neural networks to recognize rhythm stimuli from electroencephalography recordings, in Advances in Neural Information Processing Systems 27, 1449–1457.

[B49] Stober S. Prätzlich T. Müller M. (2016). Brain beats: tempo extraction from eeg data, in 17th International Society for Music Information Retrieval Conference (ISMIR'16).

[B50] Stober S. Sternin A. Owen A. M. Grahn J. A. (2015a). Deep feature learning for EEG recordings. arXiv:1511.04306.

[B51] Stober S. Sternin A. Owen A. M. Grahn J. A. (2015b). Towards music imagery information retrieval: introducing the openmiir dataset of EEG recordings from music perception and imagination, in 16th International Society for Music Information Retrieval Conference (ISMIR'15), 763–769.

[B52] Stober S. Thompson J. (2012). Music imagery information retrieval: Bringing the song on your mind back to your ears, in 13th International Conference on Music Information Retrieval (ISMIR'12) - Late-Breaking & Demo Papers.

[B53] Sturm B. L. (2014). A simple method to determine if a music information retrieval system is a “Horse.” IEEE Trans. Multimedia 16, 1636–1644. 10.1109/TMM.2014.2330697

[B54] van Merriënboer B. Bahdanau D. Dumoulin V. Serdyuk D. Warde-Farley D. Chorowski J. (2015). Blocks and fuel: frameworks for deep learning. arXiv:1506.00619.

[B55] Vincent P. Larochelle H. Lajoie I. Bengio Y. Manzagol P.-A. (2010). Stacked denoising autoencoders: learning useful representations in a deep network with a local denoising criterion. J. Mach. Learn. Res. 11, 3371–3408.

[B56] Vlek R. J. Schaefer R. S. Gielen C. C. A. M. Farquhar J. D. R. Desain P. (2011). Shared mechanisms in perception and imagery of auditory accents. Clin. Neurophysiol. 122, 1526–1532. 10.1016/j.clinph.2011.01.042 21353631

[B57] Wolff D. Weyde T. (2014). Learning music similarity from relative user ratings. Inform. Retr. 17, 109–136. 10.1007/s10791-013-9229-0

